# A Retrospective Study of Clinical and Economic Burden of Focal Segmental Glomerulosclerosis (FSGS) in the United States

**DOI:** 10.1016/j.ekir.2021.07.030

**Published:** 2021-08-09

**Authors:** Kamyar Kalantar-Zadeh, Christine L. Baker, J. Brian Copley, Daniel I. Levy, Stephen Berasi, Nihad Tamimi, Jose Alvir, Suneel M. Udani

**Affiliations:** 1 University of California Irvine, School of Medicine, Irvine, CA, USA; 2 Pfizer Inc., New York, NY, USA; 3 Medicopharma Solutions Ltd. Canterbury, Kent, UK; 4 Chicago Glomerular Disease Institute, Chicago, IL, USA

**Keywords:** claims analysis, costs, economic burden, focal segmental glomerulosclerosis, health care resource utilization, nephrotic range proteinuria

## Abstract

**Introduction:**

Information on the economic burden of focal segmental glomerulosclerosis (FSGS) is sparse. This study characterized health care resource utilization (HCRU) and costs in patients with FSGS, and evaluated the impact of nephrotic range proteinuria on these outcomes.

**Methods:**

This retrospective, observational cohort study used administrative claims data from the Optum Clinformatics Data Mart Database from October 2015 to December 2019. Patients with FSGS (*n* = 844; first claim = index event) between April 2016 and December 2018 were matched on index date, age, sex, and race to non-FSGS controls (*n* = 1688). FSGS nephrotic range (urine protein/creatinine ratio >3000 mg/g or albumin/creatinine ratio >2000 mg/g) and non-nephrotic subpopulations were identified. Baseline comorbidities, 12-month post-index all-cause HCRU and costs (per patient per year [PPPY]), and immunosuppressant prescriptions were compared between matched cohorts and between FSGS subpopulations.

**Results:**

Comorbidity burden was higher in FSGS. Of 308 patients with available urine protein/creatinine ratio/albumin/creatinine ratio results, 36.4% were in nephrotic range. All-cause HCRU was higher in FSGS across resource categories (all *P* < 0.0001); 50.6% of FSGS and 23.3% of controls were prescribed glucocorticoids *(P* < 0.0001). Mean total medical costs were higher in FSGS ($59,753 vs. $8431 PPPY; *P* < 0.0001), driven by outpatient costs. Nephrotic range proteinuria was associated with higher all-cause inpatient, outpatient, and prescription costs versus nonnephrotic patients (all *P* < 0.0001), resulting in higher total costs ($70,481 vs. $36,099 PPPY; *P* < 0.0001).

**Conclusions:**

FSGS is associated with significant clinical and economic burdens; the presence of nephrotic range proteinuria increased the economic burden. New treatment modalities are needed to reduce proteinuria, help improve patient outcomes, and reduce HCRU and associated costs.

## Graphical Abstract

**Figure:**
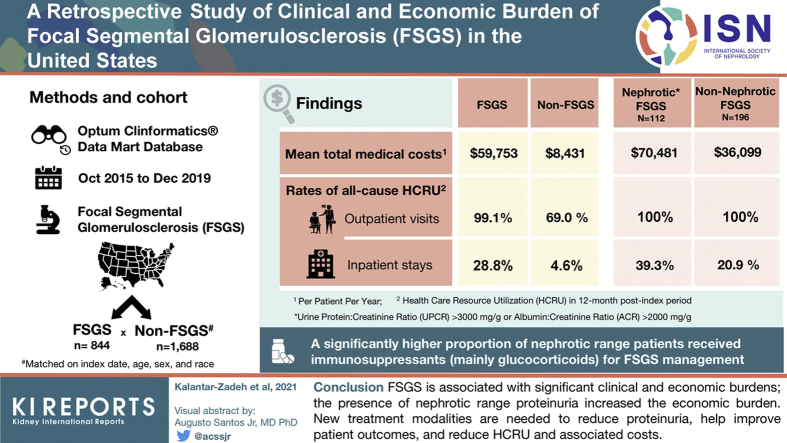

Focal segmental glomerulosclerosis (FSGS) is a common pattern of glomerular injury that may arise from diverse etiologies and often leads to proteinuric chronic kidney disease. Onset can occur at any age, and the incidence of FSGS has been estimated to be 1.4 to 21 cases per million population, with a diagnosis prevalence that has been increasing worldwide.^1^ In the United States, it is the most frequent glomerular disease that results in end-stage renal disease.^1^

Podocyte injury with progressive scarring of glomeruli is the primary pathophysiological feature, which may result in nephrotic range proteinuria or nephrotic syndrome that is considered predictive of prognosis.^2^ Patients with nephrotic syndrome have 10-year survival rates of 30% and 55%, relative to >85% for those without nephrotic syndrome, and in patients with significant proteinuria (>10 g/day), progression to end-stage renal disease occurs in 2 to 3 years on average.^2^, ^3^, ^4^, ^5^

Pharmacologic management is less than optimal. Corticosteroids are first-line treatment but result in variable remission rates and may be associated with toxicity at high dose and/or long-term use.^1^ A variety of other immunosuppressants including calcineurin inhibitors and mycophenolate mofetil may be prescribed based on the underlying pathology or evidence of steroid resistance. However, there is little evidence in support of specific recommendations for these drugs, especially for calcineurin inhibitors as first-line treatment because they may be associated with nephrotoxicity.^1^^,^^2^

The disease burden associated with FSGS has overall been poorly characterized. Limited studies have shown that FSGS is associated with impaired health-related quality of life,^6^^,^^7^ but there is a dearth of data on health care resource utilization (HCRU) and the economic burden; only a single study reported on costs associated with HCRU among patients identified with FSGS (*N* = 1187) in a commercial claims database.^8^ Although that study suggested that a small subpopulation of high utilizers of health care may drive the overall cost burden, patients were not stratified by proteinuria and thus may not accurately describe the economic burden, especially among patients with nephrotic range proteinuria. To address the gap in the economic burden of FSGS, the objective of this study was to evaluate all-cause HCRU and associated costs in patients with FSGS compared with a matched non-FSGS cohort. Additionally, the impact of nephrotic range proteinuria on HCRU and costs was evaluated.

## Methods

### Study Design and Data Sources

This retrospective, observational cohort study used administrative claims data from the Optum Clinformatics Data Mart Database for the period October 2015 to December 2019. This database accesses commercial and Medicare Advantage claims, and enrollment links patient and physician data to pharmacy and medical claims. Medical claims or encounter data, collected from all available health care sites, are deidentified such that the database is compliant with the Health Insurance Portability and Accountability Act. Institutional review board approval was not required for this study.

### Patients and Cohorts

The FSGS cohort consisted of patients who were identified based on ≥1 ICD-10-CM code for FSGS (N03.1, N04.1, N05.1, and N06.1) between April 2016 and December 2018; the first FSGS diagnosis code was considered the index event. These patients were exactly matched on age in years, sex, and race to non-FSGS patients (1:2, FSGS: non-FSGS) who did not have ICD-10-CM codes for FSGS. Matching was also conducted on index date, and potential matches were required to contain the follow-up window of the index case; if there were >2 potential matches for a FSGS case, 2 non-FSGS individuals were randomly chosen and the index date of the FSGS case was then assigned to the non-FSGS individuals. Both cohorts were required to have continuous enrollment 6 months preindex and 12 months postindex; a pre–post-index cancer diagnosis was reason for exclusion.

FSGS patients were further stratified by the presence of proteinuria within the nephrotic range and not within this range (nonnephrotic). The nephrotic range was defined as a urine protein/creatinine ratio (UPCR) >3000 mg/g or an albumin/creatinine ratio (ACR) >2000 mg/g. To avoid double counting, patients were considered in the nephrotic range if either the UPCR or the ACR value was met at any point, with identification based on an algorithm such that ACR was used if UPCR evaluation was unavailable.^9^

### Outcomes

Baseline measures were standard demographic characteristics (age, race, geographic region, income, and insurance coverage) and the presence of comorbid conditions using the Quan-Charlson Comorbidity Index (CCI).^10^ Pharmacy claims were used to determine prescription of immunosuppressant medications associated with the management of FSGS, including glucocorticoids, calcineurin inhibitors, mycophenolate mofetil, sirolimus, azathioprine, cyclophosphamide, Acthar gel, and biological agents (adalimumab, rituximab, abatacept). All-cause healthcare resource utilization was determined for resource categories of office visits, skilled nursing facilities, home health care visits, emergency department, hospitalizations, and surgeries (inpatient + outpatient). The top 10 inpatient and outpatient surgical procedures were determined in the matched cohorts based on Current Procedural Terminology codes. As an additional indicator of resource use, rates of readmission following the first inpatient hospitalization were determined at 30, 60, and 365 days in the matched cohorts.

All-cause medical costs per patient per year (PPPY) were estimated for inpatient (total of hospitalization and skilled nursing facilities), outpatient, and pharmacy costs. These costs consisted of the standard allowed charge for each category in 2019 dollars. Patient out-of-pocket (OOP) costs were estimated as the sum of coinsurance, copay, and deductible costs.

### Statistical Analysis

Study variables were analyzed using descriptive statistics. Comparison of HCRU and costs between FSGS and matched control cohorts and between nephrotic and nonnephrotic subpopulations were conducted using t tests for continuous variables and chi-square tests for categorical variables. All analyses were performed using SAS version 9.4 (SAS Institute Inc., Cary, NC) or R software (R Foundation for Statistical Computing; https://www.r-project.org/foundation/).

## Results

### Populations

Among 29,297,892 patients in the database for the study period, the 844 patients who were identified with FSGS and met all other criteria were matched with 1688 non-FSGS controls (Supplementary Table S1). In the matched cohorts, 57.4% were male, 56.9% were White, and the mean (SD) age 54.7 (18.4) years (Table 1). Significant differences were observed in other demographic characteristics including for geographic region, household income, and insurance, with the latter characterized by a higher proportion of Medicare patients in the FSGS cohort (44.8% vs. 39.5%; *P* = 0.0102).

**Table 1:** Baseline demographic characteristics of the populations

Variable	Matched cohorts			FSGS nephrotic subpopulations		
	FSGS (*n* = 844)	Non-FSGS controls (*n* = 1,688)	*P* value	Nephrotic range (*n* = 112)	Nonnephrotic (*n* = 196)	*P*-value
Male	484 (57.4)	968 (57.4)	1.00	60 (53.6)	104 (53.1)	0.9312
Age, years	54.7 ± 18.4	54.7 ± 18.4	1.00	57.0 ± 19.0	53.9 ± 17.4	0.1358
Age group			1.00			0.0537
<18 years	22 (2.6)	44 (2.6)		3 (2.7)	3 (1.5)	
18–65 years	535 (63.4)	1,070 (63.4)		60 (53.6)	133 (67.9)	
>65 years	287 (34.0)	574 (34.0)		49 (43.8)	60 (30.6)	
Race/ethnicity			1.00			0.1280
Asian	51 (6.0)	102 (6.0)		5 (4.5)	14 (7.1)	
Black	174 (20.6)	348 (20.6)		14 (12.5)	42 (21.4)	
Hispanic	139 (16.5)	278 (16.5)		29 (25.9)	38 (19.4)	
White	480 (56.9)	960 (56.9)		64 (57.1)	102 (52.0)	
Geographic region			< 0.0001			0.6527
Northeast	88 (10.4)	138 (8.2)		13 (11.6)	22 (11.2)	
Midwest	188 (22.3)	320 (19.0)		17 (15.2)	22 (11.2)	
South	405 (48.0)	717 (42.5)		60 (53.6)	104 (53.1)	
West	158 (18.7)	464 (27.5)		22 (19.6)	48 (24.5)	
Unknown	5 (0.6)	49 (2.9)				
Household income			0.0144			0.3265
<$40K	206 (24.4)	327 (19.4)		18 (16.1)	46 (23.5)	
$40K–$49K	69 (8.2)	120 (7.1)		9 (8.0)	14 (7.1)	
$50K–$59K	66 (7.8)	128 (7.6)		12 (10.7)	12 (6.1)	
$60K–$74K	75 (8.9)	164 (9.7)		13 (11.6)	17 (8.7)	
$75K–$99K	113 (13.4)	200 (11.9)		22 (19.6)	27 (13.8)	
≥$100K	185 (21.9)	446 (26.4)		23 (20.5)	48 (24.5)	
Unknown	130 (15.4)	303 (18.0)		15 (13.4)	32 (16.3)	
Insurance			0.0102			0.0938
Commercial	466 (55.2)	1,022 (60.6)		57 (50.9)	119 (60.7)	
Medicare	378 (44.8)	666 (39.5)		55 (49.1)	77 (39.3)	

FSGS, focal segmental glomerulosclerosis.

Continuous variables are expressed as mean ± SD, and categorical variables as *n* and (%).

**Figure 1:**
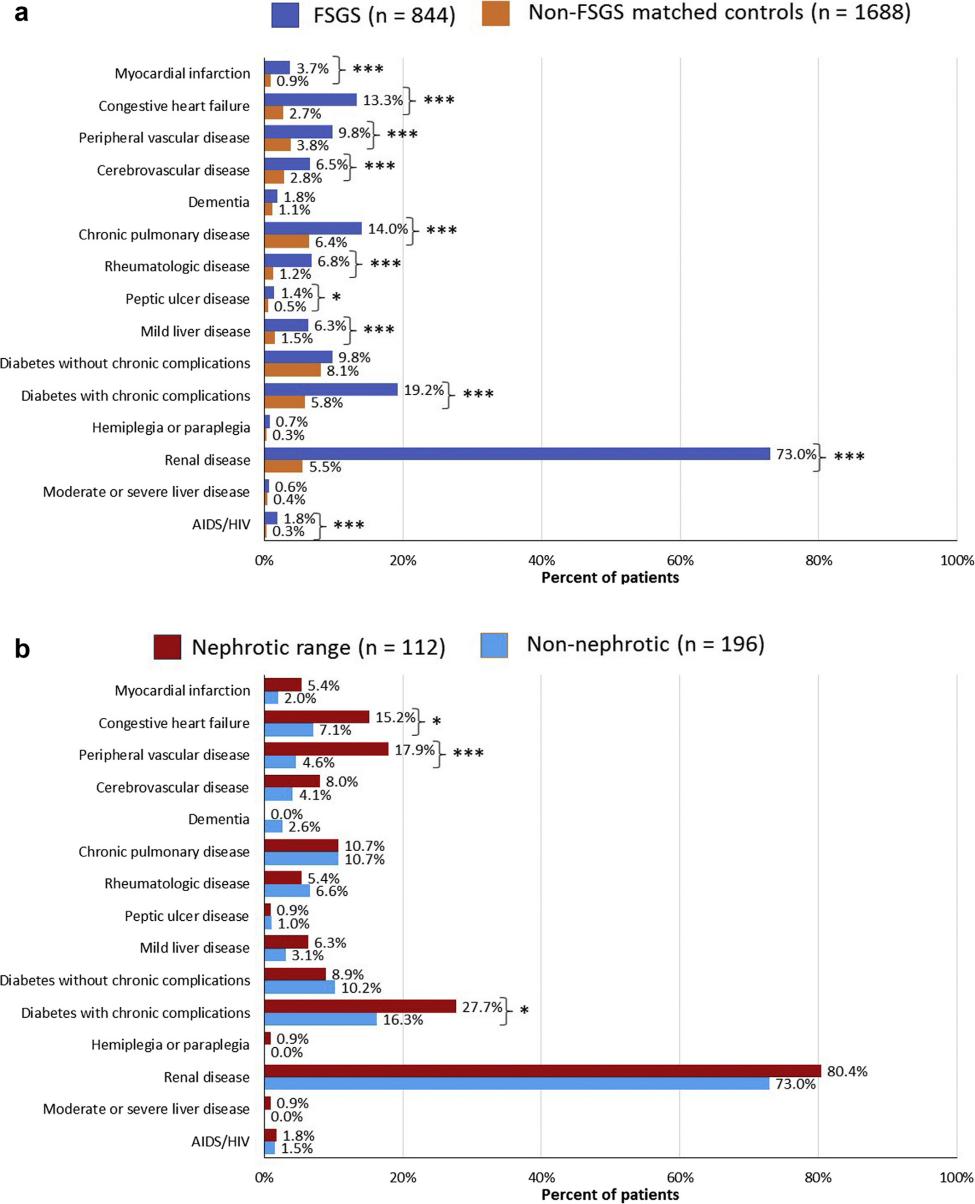
Baseline prevalence of Quan–Charlson comorbidities in (a) matched cohorts of patients with focal segmental glomerulosclerosis (FSGS) and non-FSGS controls and (b) FSGS patients with and without nephrotic range proteinuria. ∗*P* < 0.05. ∗∗∗*P* < 0.0001

The FSGS cohort had a significantly higher mean (SD) CCI (2.72 [2.12] vs. 0.55 [1.29]; *P* < 0.0001) compared with the matched controls, and a significantly higher prevalence of individual CCI comorbidities except for dementia, diabetes without chronic complications, hemiplegia/paraplegia, and moderate or severe liver disease (Figure 1a). The most prevalent CCI comorbidity in the FSGS cohort was renal disease (73.0% vs. 5.5%; *P* < 0.0001), followed by diabetes with chronic complications (19.2% vs. 5.7%; *P* < 0.0001).

In the FSGS cohort, 322 patients (38.2%) had UPCR or ACR tests during the 6-month pre- to 12-month post-index period, and among the 308 who had these results available, 112 (36.4%) were in the nephrotic range and 196 patients were nonnephrotic (63.6%). Demographic characteristics were similar between these 2 subpopulations (Table 1). However, the CCI score was significantly higher among those in the nephrotic range, 3.1 (2.1) versus 2.4 (1.7) (*P* = 0.0031), and nephrotic range patients had a significantly higher prevalence of diabetes with chronic complications, congestive heart failure, and peripheral vascular disease (all *P* < 0.05; Figure 1b).

### Health Care Resource Utilization and Costs in Matched Cohorts

**Figure 2:**
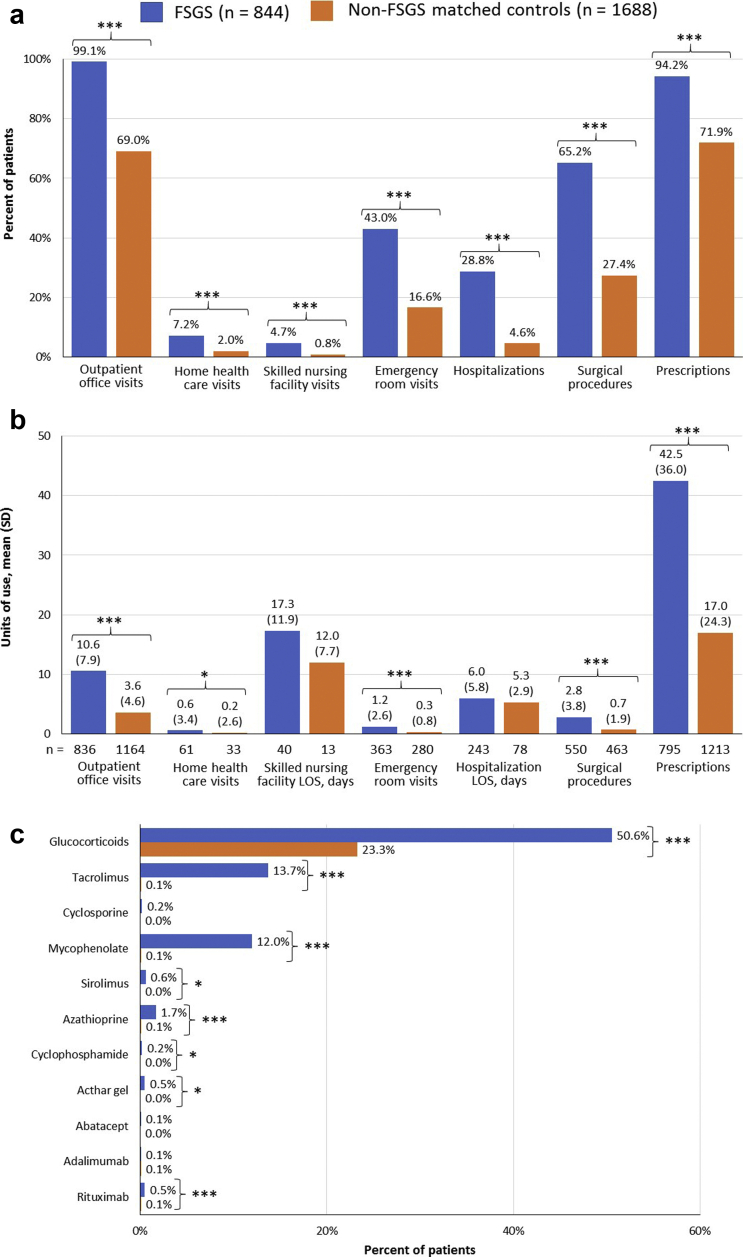
Twelve-month postindex all-cause health care resource utilization in the focal segmental glomerulosclerosis (FSGS) and matched non-FSGS control cohorts. Results are shown by (a) percent of patients with resource utilization, (b) units of resource use among those using each resource category, and (c) prescription immunosuppressants used for treatment of FSGS. ∗*P* < 0.05. ∗∗∗*P* < 0.0001. FSGS, focal segmental glomerulosclerosis; LOS, length of stay.

The FSGS cohort was characterized by significantly higher rates of all-cause HCRU in the 12-month postindex period across resource categories (all *P* < 0.0001; Figure 2a), with outpatient visits the most frequently used category (99.1% vs. 69.0%), followed by prescription medications (94.2% vs. 71.9%) and surgical procedures (65.2% vs. 27.4%). Among the patients who used these resources, units of use were significantly higher in FSGS versus matched controls for all categories except inpatient length of stay (LOS) and LOS in skilled nursing homes (Figure 2b). Medications, in particular, were heavily prescribed in the FSGS cohort, with a mean (SD) of 42.5 (36.0) prescriptions during the postindex period versus 17.0 (24.3) in the matched controls (*P* < 0.0001). Readmission rates following the first hospitalization during postindex follow-up were significantly higher in the FSGS cohort compared with matched controls at 30 days (16.1% vs. 6.0%; *P* = 0.0201) and 365 days (39.1% vs. 22.9%; *P* = 0.0073) and trended toward significance at 90 days (24.2% vs. 14.5%; *P* = 0.0628).

Among immunosuppressants that may be used for treatment of FSGS, glucocorticoids were most frequently prescribed in both the FSGS and control cohorts (Figure 2c), although the proportion of patients prescribed glucocorticoids was significantly higher in the FSGS cohort (50.6% vs. 23.3%; *P* < 0.0001). Other immunosuppressants were prescribed in <1% of controls, and of those drugs, only tacrolimus and mycophenolate mofetil were prescribed to more than 10% of FSGS patients, 13.7% and 12.0%, respectively (Figure 2c).

**Figure 3:**
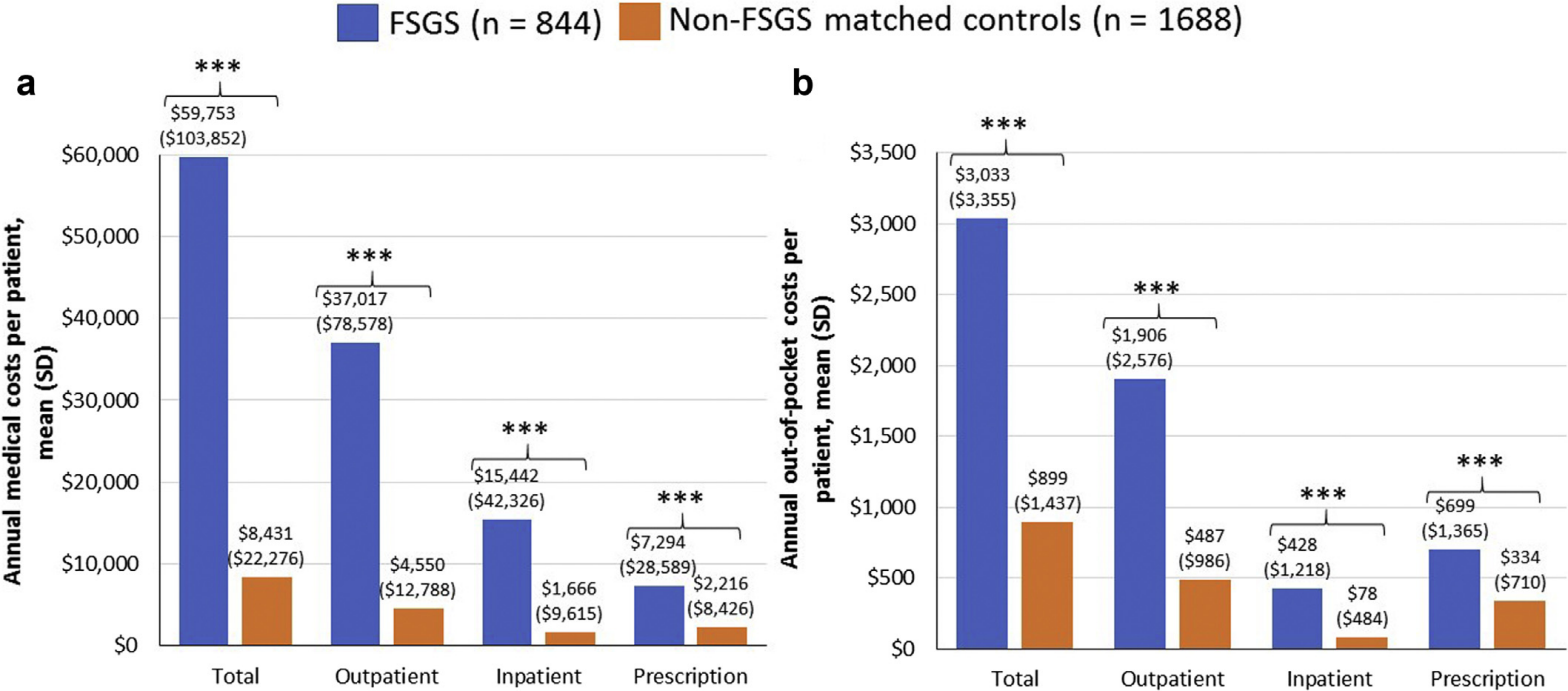
Annual health care resource utilization costs per patient including (a) medical costs and (b) patient out-of-pocket costs. ∗*P* < 0.05. ∗∗∗*P* < 0.0001. FSGS, focal segmental glomerulosclerosis.

Mean (SD) total costs were 7-fold higher in the FSGS cohort compared with matched controls ($59,753 [$103,852] vs. $8,431 [$22,276] PPPY; *P* < 0.0001) (Figure 3a). Although these costs were primarily driven by the significantly higher outpatient costs ($37,017 [$78,578] vs. $4,550 [$12,788]; *P* < 0.0001), inpatient and prescription costs were also significantly higher in the FSGS cohort (both *P* < 0.0001). Patients with FSGS incurred higher outpatient, inpatient, and prescription OOP costs vs. matched controls (all *P* < 0.0001; Figure 3b), resulting in mean total OOP costs that were more than 3-fold higher ($3,033 [$3,355] vs. $899 [$1,437] PPPY; *P* < 0.0001).

### Health Care Resource Utilization and Costs in Nephrotic Range Subpopulation

The presence of nephrotic range proteinuria was associated with higher all-cause HCRU and associated costs (Table 1 and Figure 3). Regardless of the presence of nephrotic proteinuria, all patients had at least 1 outpatient office visit during the 12-month postindex follow-up period (Table 2). However, the mean number of visits was significantly higher among patients within the nephrotic range (14.6 [8.7] vs. 10.0 [7.7]; *P* < 0.0001). Both the rate and the units of emergency department use were significantly higher among those within the nephrotic range (*P* < 0.05), and a higher proportion of patients with nephrotic range proteinuria had inpatient stays (39.3% vs. 20.9%; *P* = 0.0005), but there was no difference in LOS (Table 2). The proportion of patients with any medication prescription was similarly high in the 2 groups, but those within the nephrotic range had a significantly higher mean number of prescriptions (49.0 [32.5] vs. 39.7 [31.5; *P* = 0.0141) (Table 2).

**Table 2:** Health care resource utilization during 1 year of postindex follow-up among patients with focal segmental glomerulosclerosis with and without nephrotic range proteinuria

Variable	Nephrotic range (*n* = 112)	Non-nephrotic (*n* = 196)	*P* value
Outpatient office visits			
Patients with visits	112 (100)	196 (100)	—
Number of visits	14.6 ± 8.7	10.0 ± 7.7	<0.0001
Emergency department visits			
Patients with visits	56 (50.0)	70 (35.7)	0.0142
Number of visits	1.5 ± 4.1	0.7 ± 1.3	0.0476
Inpatient stays			
Patients with stays, *n* (%)	44 (39.3)	41 (20.9)	0.0005
Length of stay, mean ± SD	5.7 ± 4.5	5.9 ± 4.0	0.7930
Prescriptions			
Patients with prescriptions, *n* (%)	109 (97.3)	187 (95.4)	0.4039
Number of prescriptions, mean ± SD	49.0 ± 32.5	39.7 ± 31.5	0.0141
FSGS medication prescriptions, *n* (%)			0.0106
No disease-modifying drugs	30 (26.8)	90 (45.9)	
Glucocorticoids only	52 (46.4)	65 (33.2)	
Glucocorticoids + other disease-modifying drugs	23 (20.5)	33 (16.8)	
Nonglucocorticoids	7 (6.3)	8 (4.1)	
Any glucocorticoid, *n* (%)	75 (67.0)	98 (50.0)	0.0039

FSGS, focal segmental glomerulosclerosis.

Continuous variables are expressed as mean ± SD, and categorical variables as *n* and (%).

Although a significantly higher proportion of nephrotic range patients were prescribed immunosuppressant medications associated with the management of FSGS (73.2% vs. 54.1%; *P* = 0.0009), more than one-quarter of the nephrotic range group (26.8%) were not prescribed any of these drugs during the follow-up period (Table 2). Glucocorticoids, mainly as monotherapy, were the most frequently prescribed immunosuppressant in both groups; low proportions of patients were prescribed nonglucocorticoid immunosuppressants (Table 2).

**Figure 4:**
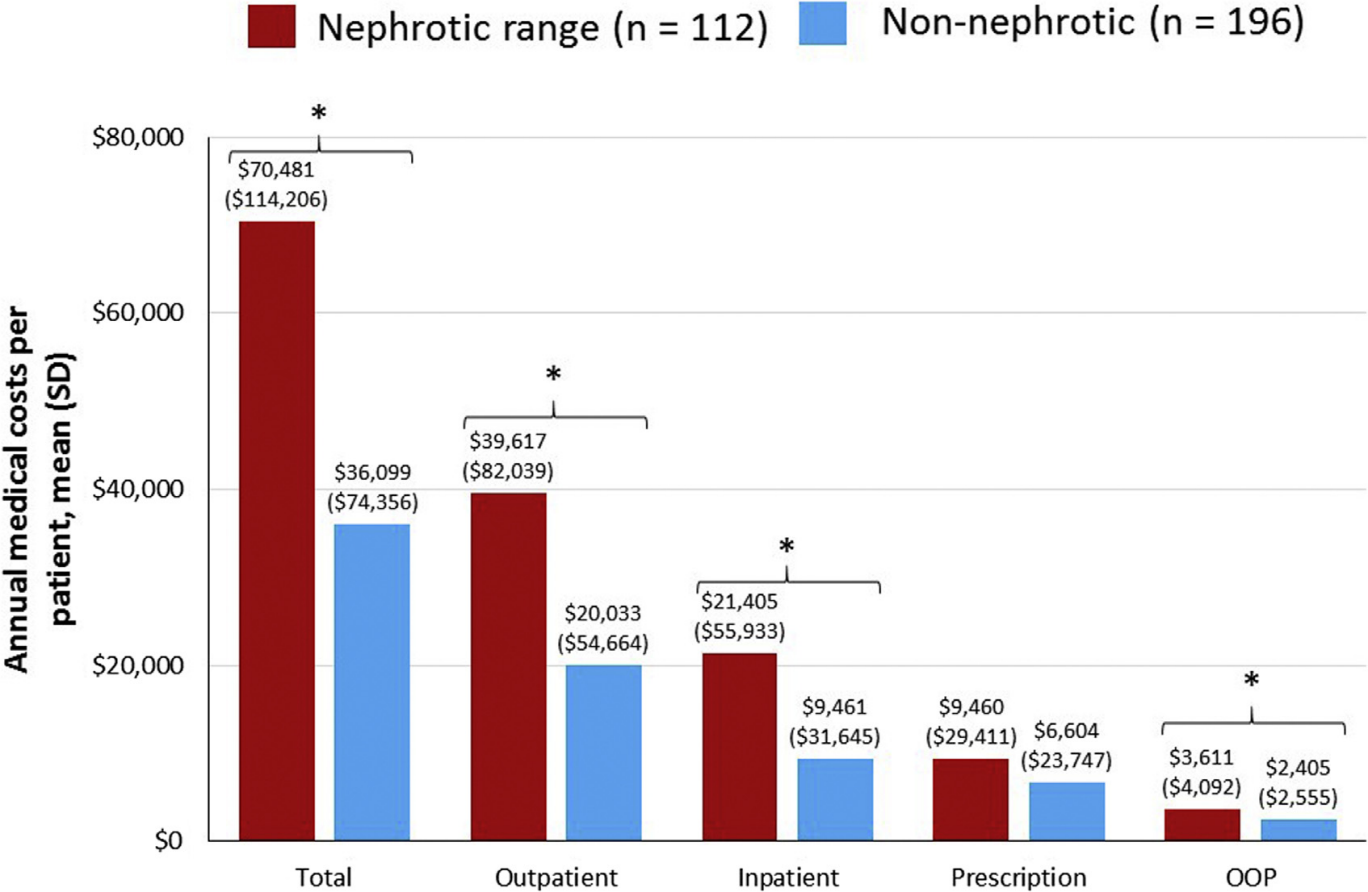
Annual health care resource utilization costs per patient among patients with FSGS with and without nephrotic range proteinuria. ∗*P* < 0.05. ∗∗∗*P* < 0.0001. FSGS, focal segmental glomerulosclerosis; OOP, out of pocket; SD, standard deviation.

Mean (SD) total all-cause medical costs were $70,481 ($114,206) PPPY in patients with nephrotic proteinuria versus $36,099 ($74,356) PPPY in the nonnephrotic group (*P* = 0.0048), primarily driven by a outpatient costs (Figure 4). Nephrotic range proteinuria was associated with approximately 2-fold higher inpatient and outpatient costs versus nonnephrotic patients (both *P* < 0.0001), although prescription costs were similar in the 2 groups (Figure 4). Patients in both groups incurred substantial OOP costs, which were significantly higher among those in the nephrotic range ($3,611 [$4,092] vs. $2,405 [$2,555]; *P* = 0.0054).

### Surgical Procedures

A more detailed analysis to identify the cause of the high rate of surgical procedures showed that 522 outpatient and 150 inpatient surgical procedures were performed in the FSGS cohort; in the matched controls, 453 and 39 outpatient and inpatient surgeries were performed, respectively. The primary outpatient procedure among FSGS patients was renal biopsy, which accounted for 54.4% of these surgeries (Supplementary Table S2). In contrast, the most frequent procedure among the matched controls, arthrocentesis and related procedures, accounted for only 18.5% of outpatient procedures. However, there was some overlap between cohorts in outpatient surgical procedures (Supplementary Table S2).

Three of the top 10 inpatient and outpatient surgeries in FSGS patients were renal procedures. The most frequent inpatient surgical procedure in FSGS patients was insertion of a tunneled centrally inserted central venous catheter, accounting for 31.3% of these procedures, whereas catheter placement in a coronary artery for coronary angiography was the primary procedure in matched controls (18.0% of procedures) (Supplementary Table S3).

## Discussion

This is the first study to comprehensively compare clinical and economic burdens of FSGS with a matched control cohort and to evaluate the impact of nephrotic range proteinuria on the economic burden. The results provide evidence for a higher comorbidity burden, greater HCRU, and a substantially higher economic burden among patients with FSGS. Importantly, the presence of nephrotic range proteinuria conveyed an additional burden as indicated by higher HCRU and costs relative to patients with FSGS who were nonnephrotic.

After matching, a significant difference was observed between FSGS and non-FSGS cohorts with regard to insurance type, driven by a higher proportion of FSGS patients on Medicare that likely reflects eligibility due to disability. This disability may result not only from FSGS, but also likely reflects the higher clinical burden with regard to more prevalent diagnoses including pulmonary, cardiovascular, rheumatologic, and gastrointestinal conditions. The prevalence of several comorbidities, including diabetes and cardiovascular disease, was consistent with a previous report.^11^ These conditions, which have been shown to increase the costs associated with chronic kidney disease,^12^ are also likely to have a reciprocal relationship with FSGS and need to be considered when initiating treatment.^1^^,^^13^^,^^14^

Compared with matched controls, FSGS was associated with higher rates of HCRU across all resource categories, and higher units of resource use that were significant except for LOS in the hospital and skilled nursing home settings. However, the standard deviations were high across categories, suggesting that patients have a range of severity that is not captured in claims databases. Outpatient visits was the most frequently used category, and prescriptions represented the most heavily used category by units of use, likely also reflecting the higher comorbidity burden. Although it is not surprising that immunosuppressant prescriptions were higher in the FSGS cohort, it should be noted that these medications may be prescribed for other conditions.

Among immunosuppressants, glucocorticoids were most frequently prescribed, consistent with clinical practice.^1^^,^^13^^,^^14^ The reliance on glucocorticoids may itself increase costs of care due to their well-recognized adverse effects. Also consistent with clinical practice was the use of second- and third-line immunosuppressants, which may be indicative of the failure of glucocorticoids to achieve adequate disease control. However, there are few supporting studies for providing treatment recommendations, and use of therapies is also dependent on the underlying FSGS etiology and susceptibility/resistance to steroid therapy, neither of which are captured in this database.

Readmissions after the first postindex hospitalization were substantial in the FSGS cohort, with more than one-third of the patients (39.1%) readmitted within the year. This high rate is relevant not only from the economic perspective, because hospitalizations are the resource category with the highest unit cost, but also from the clinical perspective due to inadequate disease control and progressive complications from the disease itself as well as from side effects of current interventions. However, whether the hospitalizations were due to FSGS or another condition was not specifically evaluated. Surgery, in particular, had a high rate of utilization in the FSGS cohort, although overlap of several procedures between the FSGS and non-FSGS cohorts may reflect matching of age and sex. FSGS surgeries were predominantly driven by outpatient procedures that were not necessarily directly related to FSGS. While this suggests a substantial surgical burden borne by these patients that contributes to overall costs, it should be noted that the majority of procedures were for renal biopsy, which is the entry point for a diagnosis of FSGS. Another common procedure was placement of dialysis access, further supporting continuation of the high costs associated with care as patients progress to dialysis and end-stage renal disease.

Consistent with the higher HCRU relative to the matched controls, there was a substantial economic burden, with significantly higher inpatient, outpatient, and prescription costs that resulted in higher annual total costs. The estimated total annual costs of $59,793 per patient with FSGS were slightly higher than a similar study,^8^ which reported mean (SD) annual per patient costs of $44,397 ($102,482), and may be accounted for by the more recent time period encompassed by the current study. Although that study also identified a small subpopulation (5%) of high-cost patients, such a subpopulation was not specifically characterized in the current analysis. However, the large standard deviations in the current analysis suggest that some patients were high utilizers of HCRU and had associated higher costs. In contrast to that study, the current analysis stratified by the presence of nephrotic proteinuria and showed that the annual cost of these patients, $70,481, had costs that were substantially higher than the overall FSGS population and significantly higher than nonnephrotic patients. For additional context, these costs approach those associated with hemodialysis among Medicare patients with end-stage renal disease ($91,795 per patient per year), and higher than the annual costs of maintaining a renal transplant patient ($35,817).^12^

Inpatient costs represent the highest cost-per-event resource category, yet it only accounted for one-quarter (25.8%) of total costs. Outpatient costs were the primary cost driver, 62.0% of total costs, similar to the previous study that also identified this resource category as the cost driver (57.2% of total costs).^8^ The patients themselves incurred a substantial economic burden, with OOP costs of $3,033 that were significantly higher than matched controls.

Although nephrotic range proteinuria may be associated with poorer outcomes, especially if untreated, this study also shows that it is associated with a significantly higher economic burden resulting from greater HCRU. Of greater clinical relevance may be the fact that only 38.2% of FSGS patients had UPCR or ACR data available during the study period. This low proportion may reflect inadequate monitoring, suggesting the need for more frequent assessment; however, it is also possible that, at least in some cases, testing occurred outside of the health care system captured by this database.

These claims data broadly outline the natural history of many patients with FSGS, from biopsy, which was the primary surgical procedure, to treatment including use of second- and third-line agents generally indicative of lack of response to first-line agents, to progression to advanced kidney disease and the potential need for dialysis. Throughout this course, patients have used more health care resources and incurred a higher economic burden yet still arrive at a less than optimal outcome (dialysis dependence or need for transplant), suggesting the need for additional therapeutic options for patient management.

### Limitations

Several limitations associated with claims databases should be noted, including the potential for misclassification; lack of clinical information, such as disease severity or progression that likely contribute to resource use and costs; and inability to differentiate primary versus secondary FSGS, which may be treated differently and thus may be associated with differences in HCRU and costs. Claims data also cannot directly link resource use to the disease of interest, which is especially relevant regarding medication prescriptions, nor can it be determined whether the prescriptions were filled or used as prescribed. Another limitation is that because kidney transplant patients were not excluded, the likely higher presence of such patients in the FSGS cohort may have accounted for the higher use of some immunosuppressants. It should also be noted in regard to transplant patients that some of their costs may be related to posttransplant complications, potentially overestimating the cost burden. However, the presence of FSGS in these patients warranted their inclusion because they nevertheless contribute to the cost burden aside from outcomes that may be due to the transplantation itself. Finally, neither indirect costs, such as those related to lost productivity, nor utility costs associated with impaired quality of life were evaluated, suggesting a need for broader assessment of the socioeconomic impact of FSGS.

In summary, this study provides a current assessment of the high medical and economic burdens associated with FSGS, with the economic burden in particular resulting in total medical costs that are more than 7-fold higher than a matched non-FSGS cohort. Although outpatient costs appear to be the major cost driver, the presence of nephrotic proteinuria substantially and significantly increases the economic burden. The reliance on glucocorticoids and lack of alternative options for pharmacologic management suggests the need for new treatment modalities that may help improve patient outcomes while reducing HCRU and associated costs.

## Disclosures

KK-Z has received honoraria and/or support from Abbott, Abbvie, ACI Clinical (Cara Therapeutics), Akebia, Alexion, Amgen, Ardelyx, American Society of Nephrology, Astra-Zeneca, Aveo, BBraun, Chugai, Cytokinetics, Daiichi, DaVita, Fresenius, Genentech, Haymarket Media, Hofstra Medical School, International Federation of Kidney Foundations, International Society of Hemodialysis, International Society of Renal Nutrition & Metabolism, Japanese Society of Dialysis Therapy, Hospira, Kabi, Keryx, Kissei, Novartis, OPKO, NIH, National Kidney Foundations, Pfizer, Regulus, Relypsa, Resverlogix, Dr Schaer, Sandoz, Sanofi, Shire, Veterans’ Affairs, Vifor, UpToDate, and ZS-Pharma. SMU has received honoraria and/or support from Abbot, Alexion, Aurinia Boehringer-Ingelheim, Natera, Omeros, Pfizer and Retrophin. JBC and NT are independent consultants currently doing contracting work for Pfizer. CLB, DIL, SB, and JA are employees and stockholders of Pfizer Inc., the sponsor of this study.

## Supplementary Material

Supplementary File (PDF)

**Table S1.** Cohort attrition.

**Table S2.** The 10 most frequent outpatient surgeries identified using Clinical Procedure Terminology (CPT) codes.

**STROBE Statement**

## References

[1] Shabaka A., Tato Ribera A., Fernandez-Juarez G. (2020). Focal segmental glomerulosclerosis: state-of-the-art and clinical perspective. Nephron.

[2] Cattran D.C., Appel G.B. (2019). Focal segmental glomerulosclerosis: Treatment of primary focal segmental glomerulosclerosis. UpTpoDate.

[3] Cameron J.S., Turner D.R., Ogg C.S., Chantler C., Williams D.G. (1978). The long-term prognosis of patients with focal segmental glomerulosclerosis. Clin Nephrol.

[4] Velosa J.A., Holley K.E., Torres V.E., Offord K.P. (1983). Significance of proteinuria on the outcome of renal function in patients with focal segmental glomerulosclerosis. Mayo Clin Proc.

[5] Rydel J.J., Korbet S.M., Borok R.Z., Schwartz M.M. (1995). Focal segmental glomerular sclerosis in adults: presentation, course, and response to treatment. Am J Kidney Dis.

[6] Canetta P.A., Troost J.P., Mahoney S. (2019). Health-related quality of life in glomerular disease. Kidney Int.

[7] Troost J.P., Waldo A., Carlozzi N.E. (2020). The longitudinal relationship between patient-reported outcomes and clinical characteristics among patients with focal segmental glomerulosclerosis in the Nephrotic Syndrome Study Network. Clin Kidney J.

[8] Nazareth T.A., Kariburyo F., Kirkemo A. (2017). Patients with focal segmental glomerulosclerosis (FSGS): a claims analysis of clinical and economic outcomes [abstract TH-PO168]. J Am Soc Nephrol.

[9] Weaver R.G., James M.T., Ravani P. (2020). Estimating urine albumin-to-creatinine ratio from protein-to-creatinine ratio: development of equations using same-day measurements. J Am Soc Nephrol.

[10] Quan H., Sundararajan V., Halfon P. (2005). Coding algorithms for defining comorbidities in ICD-9-CM and ICD-10 administrative data. Med Care.

[11] O’Shaughnessy M.M., Montez-Rath M.E., Lafayette R.A., Winkelmayer W.C. (2015). Patient characteristics and outcomes by GN subtype in ESRD. Clin J Am Soc Nephrol.

[12] System USRD (2019). https://www.usrds.org/media/2371/2019-executive-summary.pdf.

[13] Rovin B.H., Caster D.J., Cattran D.C. (2019). Management and treatment of glomerular diseases (part 2): conclusions from a Kidney Disease: Improving Global Outcomes (KDIGO) Controversies Conference. Kidney Int.

[14] Floege J., Barbour S.J., Cattran D.C. (2019). Management and treatment of glomerular diseases (part 1): conclusions from a Kidney Disease: Improving Global Outcomes (KDIGO) Controversies Conference. Kidney Int.

