# Supplementary material for: A Retrospective Study of Clinical and Economic Burden of Focal Segmental Glomerulosclerosis (FSGS) in the United States
Source: Kidney Int Rep. 2021 Aug 9;6(10):2679–88. doi: 10.1016/j.ekir.2021.07.030 (PMC8484118; doi:10.1016/j.ekir.2021.07.030)
Supplement: Supplementary File (PDF) [file mmc1.pdf]

Supplementary Table S1. Cohort attrition

Cohort Selection Step	FSGS	Non-FSGS
1. All patients enrolled in Optum SES commercial or Medicare April 2016 to December 2018	29,297,892	29,297,892
2. With/without any FSGS diagnosis	4,289	29,292,389
3. Pre-match on year of birth, gender, and race	—	17,683,093
4. With 6 months pre-index continuous enrollment	2,215	11,201,071
5. With 12 months post-index continuous enrollment	1,639	8,368,881
6. With no cancer diagnosis during 6 months pre-index through 12 months post-index	935	6,572,015
7. With matched control patients	844	1,688

FSGS, focal segmental glomerulosclerosis

Supplementary Table S2. The 10 most frequent outpatient surgeries identified using Clinical Procedure Terminology (CPT) codes

FSGS (n = 522)			Matched controls (n = 453)		
**CPT code**	**Procedure**	**Number (%)**	**CPT code**	**Procedure**	**Number (%)**
50200	Renal biopsy; percutaneous, by trocar or needle	284 (54.4)	20610	Arthrocentesis, aspiration and/or injection, major joint or bursa (eg, shoulder, hip, knee, subacromial bursa); without ultrasound guidance	84 (18.5)
20610	Arthrocentesis, aspiration and/or injection, major joint or bursa (eg, shoulder, hip, knee, subacromial bursa); without ultrasound guidance	98 (18.8)	66984	Extracapsular cataract removal with insertion of intraocular lens prosthesis (1 stage procedure), manual or mechanical technique (eg, irrigation and aspiration or phacoemulsification)	73 (16.1)
36902	Introduction of catheter dialysis circuit	84 (16.1)	11721	Debridement of nail(s) by any method(s); 6 or more	51 (11.3)
11721	Debridement of nail(s) by any method(s); 6 or more	77 (14.8)	67028	Intravitreal injection of a pharmacologic agent (separate procedure)	51 (11.3)
36821	Arteriovenous anastomosis, open; direct, any site (eg, cimino type) (separate procedure)	72 (13.8)	51798	Measurement of post-voiding residual urine and/or bladder capacity by ultrasound, non-imaging	43 (9.5)
51798	Measurement of post-voiding residual urine and/or bladder capacity by ultrasound, non-imaging	72 (13.8)	11042	Debridement, subcutaneous tissue (includes epidermis and dermis, if performed); first 20 sq cm or less	41 (9.1)
36589	Removal of tunneled central venous catheter, without subcutaneous port or pump	69 (13.2)	43239	Esophagogastroduodenoscopy, flexible, transoral; with biopsy, single or multiple	38 (8.4)
67028	Intravitreal injection of a pharmacologic agent (separate procedure)	61 (11.7)	69210	Removal impacted cerumen requiring instrumentation, unilateral	33 (7.3)

FSGS (n = 522)			Matched controls (n = 453)		
**CPT code**	**Procedure**	**Number (%)**	**CPT code**	**Procedure**	**Number (%)**
11042	Debridement, subcutaneous tissue (includes epidermis and dermis, if performed); first 20 cm^2 or less	40 (7.7)	45378	Colonoscopy, flexible; diagnostic, including collection of specimen(s) by brushing or washing, when performed (separate procedure)	31 (6.8)
66984	Extracapsular cataract removal with insertion of intraocular lens prosthesis (1 stage procedure), manual or mechanical technique (eg, irrigation and aspiration or phacoemulsification)	40 (7.7)	17000	Destruction (eg, laser surgery, electrosurgery, cryosurgery, chemosurgery, surgical curettement), premalignant lesions (eg, actinic keratoses); first lesion	21 (4.6)

Supplementary Table S3. The 10 most frequent inpatient surgeries identified using Clinical Procedure Terminology (CPT) codes

FSGS (n = 150)			Matched controls (n = 39)		
**CPT code**	**Procedure**	**Number (%)**	**CPT code**	**Procedure**	**Number (%)**
36558	Insertion of tunneled centrally inserted central venous catheter, without subcutaneous port or pump; age 5 years or older	47 (31.3)	93458	Catheter placement in coronary artery(s) for coronary angiography, including intraprocedural injection(s) for coronary angiography, imaging supervision and interpretation; with left heart catheterization including intraprocedural injection(s) for left ventriculography, when performed	7 (18.0)
36556	Insertion of non-tunneled centrally inserted central venous catheter; age 5 years or older	21 (14.0)	36556	Insertion of non-tunneled centrally inserted central venous catheter; age 5 years or older	4 (10.3)
50360	Renal allotransplantation, implantation of graft; without recipient nephrectomy	20 (13.3)	92928	Percutaneous transcatheter placement of intracoronary stent(s), with coronary angioplasty when performed; single major coronary artery or branch	4 (10.3)
43239	Esophagogastroduodenoscopy, flexible, transoral; with biopsy, single or multiple	14 (9.3)	22853	Insertion of interbody biomechanical device(	3 (7.7)
93458	Catheter placement in coronary artery(s) for coronary angiography, including intraprocedural injection(s) for coronary angiography, imaging supervision and interpretation; with left heart catheterization including intraprocedural injection(s) for left ventriculography, when performed	12 (8.0)	27447	Arthroplasty, knee, condyle and plateau; medial and lateral compartments with or without patella resurfacing (total knee arthroplasty)	3 (7.7)

FSGS (n = 150)			Matched controls (n = 39)		
**CPT code**	**Procedure**	**Number (%)**	**CPT code**	**Procedure**	**Number (%)**
27447	Arthroplasty, knee, condyle and plateau; medial and lateral compartments with or without patella resurfacing (total knee arthroplasty)	10 (6.7)	36558	Insertion of tunneled centrally inserted central venous catheter, without subcutaneous port or pump; age 5 years or older	3 (7.7)
50323	Backbench standard preparation of cadaver donor renal allograft prior to transplantation, including dissection and removal of perinephric fat, diaphragmatic and retroperitoneal attachments, excision of adrenal gland, and preparation of ureter(s), renal vein(s), and renal artery(s), ligating branches, as necessary	10 (6.7)	44602	Suture of small intestine (enterorrhaphy) for perforated ulcer, diverticulum, wound, injury or rupture; single perforation	3 (7.7)
50200	Renal biopsy; percutaneous, by trocar or needle	9 (6.0)	63047	Laminectomy, facetectomy and foraminotomy (unilateral or bilateral with decompression of spinal cord, cauda equina and/or nerve root[s], [eg, spinal or lateral recess stenosis]), single vertebral segment; lumbar	3 (7.7)
36620	Arterial catheterization or cannulation for sampling, monitoring or transfusion (separate procedure); percutaneous	8 (5.3)	92941	Percutaneous transluminal revascularization of acute total/subtotal occlusion during acute myocardial infarction, coronary artery or coronary artery bypass graft, any combination of intracoronary stent, atherectomy and angioplasty, including aspiration thrombectomy when performed, single vessel	3 (7.7)

FSGS (n = 150)			Matched controls (n = 39)		
**CPT code**	**Procedure**	**Number (%)**	**CPT code**	**Procedure**	**Number (%)**
50547	Laparoscopy, surgical; donor nephrectomy (including cold preservation), from living donor	8 (5.3)	93454	Catheter placement in coronary artery(s) for coronary angiography, including intraprocedural injection(s) for coronary angiography, imaging supervision and interpretation	3 (7.7)

STROBE Statement—checklist of items that should be included in reports of observational studies

	Item No	Recommendation	Page No
**Title and abstract**	1	(*a*) Indicate the study's design with a commonly used term in the title or the abstract	1
		(*b*) Provide in the abstract an informative and balanced summary of what was done and what was found	2
**Introduction**			
Background/rationale	2	Explain the scientific background and rationale for the investigation being reported	4-5
Objectives	3	State specific objectives, including any prespecified hypotheses	5
**Methods**			
Study design	4	Present key elements of study design early in the paper	5-6
Setting	5	Describe the setting, locations, and relevant dates, including periods of recruitment, exposure, follow-up, and data collection	5-6
Participants	6	(*a*) *Cohort study*—Give the eligibility criteria, and the sources and methods of selection of participants. Describe methods of follow-up<br>*Case-control study*—Give the eligibility criteria, and the sources and methods of case ascertainment and control selection. Give the rationale for the choice of cases and controls<br>*Cross-sectional study*—Give the eligibility criteria, and the sources and methods of selection of participants	5-6
		(*b*) *Cohort study*—For matched studies, give matching criteria and number of exposed and unexposed<br>*Case-control study*—For matched studies, give matching criteria and the number of controls per case	5-6
Variables	7	Clearly define all outcomes, exposures, predictors, potential confounders, and effect modifiers. Give diagnostic criteria, if applicable	6-7
Data sources/ measurement	8*	For each variable of interest, give sources of data and details of methods of assessment (measurement). Describe comparability of assessment methods if there is more than one group	6-7
Bias	9	Describe any efforts to address potential sources of bias	Not applicable
Study size	10	Explain how the study size was arrived at	Not applicable
Quantitative variables	11	Explain how quantitative variables were handled in the analyses. If applicable, describe which groupings were chosen and why	6-7
Statistical methods	12	(*a*) Describe all statistical methods, including those used to control for confounding	7
		(*b*) Describe any methods used to examine subgroups and interactions	Not applicable

		(*c*) Explain how missing data were addressed	Not applicable
		(*d*) *Cohort study*—If applicable, explain how loss to follow-up was addressed<br><br>*Case-control study*—If applicable, explain how matching of cases and controls was addressed<br><br>*Cross-sectional study*—If applicable, describe analytical methods taking account of sampling strategy	Not applicable
		(*e*) Describe any sensitivity analyses	

**Results**			
Participants	13*	(a) Report numbers of individuals at each stage of study—eg numbers potentially eligible, examined for eligibility, confirmed eligible, included in the study, completing follow-up, and analysed	Table S1
		(b) Give reasons for non-participation at each stage	Not applicable
		(c) Consider use of a flow diagram	
Descriptive data	14*	(a) Give characteristics of study participants (eg demographic, clinical, social) and information on exposures and potential confounders	Table 1
		(b) Indicate number of participants with missing data for each variable of interest	Not applicable
		(c) *Cohort study*—Summarise follow-up time (eg, average and total amount)	Not applicable
Outcome data	15*	*Cohort study*—Report numbers of outcome events or summary measures over time	7-11; figures and tables
		Case-control study—Report numbers in each exposure category, or summary measures of exposure	
		Cross-sectional study—Report numbers of outcome events or summary measures	
Main results	16	(*a*) Give unadjusted estimates and, if applicable, confounder-adjusted estimates and their precision (eg, 95% confidence interval). Make clear which confounders were adjusted for and why they were included	Not applicable
		(*b*) Report category boundaries when continuous variables were categorized	Not applicable
		(*c*) If relevant, consider translating estimates of relative risk into absolute risk for a meaningful time period	Not applicable
Other analyses	17	Report other analyses done—eg analyses of subgroups and interactions, and sensitivity analyses	Not applicable
**Discussion**			
Key results	18	Summarise key results with reference to study objectives	12
Limitations	19	Discuss limitations of the study, taking into account sources of potential bias or imprecision. Discuss both direction and magnitude of any potential bias	16

Interpretation	20	Give a cautious overall interpretation of results considering objectives, limitations, multiplicity of analyses, results from similar studies, and other relevant evidence	15-16
Generalisability	21	Discuss the generalisability (external validity) of the study results	15-16
**Other information**			
Funding	22	Give the source of funding and the role of the funders for the present study and, if applicable, for the original study on which the present article is based	18

*Give information separately for cases and controls in case-control studies and, if applicable, for exposed and unexposed groups in cohort and cross-sectional studies.

Note: An Explanation and Elaboration article discusses each checklist item and gives methodological background and published examples of transparent reporting. The STROBE checklist is best used in conjunction with this article (freely available on the Web sites of PLoS Medicine at http://www.plosmedicine.org/, Annals of Internal Medicine at http://www.annals.org/, and Epidemiology at http://www.epidem.com/). Information on the STROBE Initiative is available at www.strobe-statement.org.
